# Study on Mechanical Characteristics of Segmental Joints of a Large-Diameter Shield Tunnel under Ultrahigh Water Pressure

**DOI:** 10.3390/s21248392

**Published:** 2021-12-16

**Authors:** Lei Kou, Zhihui Xiong, Hao Cui, Jinjie Zhao

**Affiliations:** School of Water Conservancy Science and Engineering, Zhengzhou University, Zhengzhou 450001, China; Xzhihui1998@163.com (Z.X.); cuiho831@163.com (H.C.); zhaojinjie666@163.com (J.Z.)

**Keywords:** high water pressure, shield tunnel, segmental joints, theoretical model, numerical simulation

## Abstract

At present, there is no clear design standard for segmental joints of large-diameter shield tunnels under high water pressure. In this paper, a theoretical calculation model for the bending stiffness of segmental joints under high water pressure is proposed. The numerical simulation method is used to investigate the failure and crack formation processes of single-layer and double-layer lining segments under large axial forces. The effects of axial force, bolt strength, and concrete strength on the bending stiffness of joints are then studied using a theoretical calculation model of segmental joints. The results show that under extremely high water pressure, the influence of double lining on joint stiffness is limited. It is more rational and safe to compute the bending stiffness of segmental joints using this theoretical model rather than the numerical simulation method. The parameter analysis reveals that increasing the bolt strength has a minor impact on bending stiffness and deformation, whereas increasing the concrete strength has the opposite effect. The influence of ultimate bearing capacity and deformation decreases non-linearly as the axial force increases.

## 1. Introduction

Tunnels play an essential role in the transportation infrastructure. For example, traffic tunnels such as roads or railways are built under the sea or rivers to speed regional connections and economic development in China, mainly using the large-diameter shield construction technology.

The shield tunnel lining structure consists of segments assembled into rings through joints. In the actual working state, the segmental joint is subjected to a combination of axial force, shear force, and bending moment. The mechanical behavior of the concrete joint shows complex nonlinearity, which affects the mechanical properties of shield tunnel lining structures. Therefore, the mechanical characteristics of segmental joints have always been a vital issue and important research topic in shield tunnel design.

Previous studies [1,2,3,4] have shown that the joint and the segment are discontinuous, and the bending stiffness of the joint is lower than that of the segment, making the joint the weakest part of the structure. Therefore, the internal force analysis of the shield structure must consider the influence of the joint on the stiffness reduction of the overall segment.

The joint stiffness is related to the bearing capacity of the shield tunnel design and the operation performance under normal operating conditions. Especially for subsea tunnels under high water pressure, the bending stiffness of the joint directly affects the waterproof performance of the tunnel and thus affect the operation and maintenance of the whole tunnel.

The calculation model of segmental joints without lining was proposed by Murakami and Koizumi [5]. The model only considered concrete stress in the compression zone at the joint surface and the stress of the bolts. Moreover, the model adopted the plane section assumption, which can better consider the stress distribution of the concrete joint surface and the stress of the bolts. The model initially assumed that the resultant force point of the compression zone was fixed to derive a fixed value of the joint flexural stiffness. After that, Inftimie [6] improved the model and considered that the compressive deformation of concrete was the main influencing factor of joint deformation. However, this model did not consider the joint bearing effect of concrete compression reinforcement and the situation that the concrete in the compression zone did not meet the plane section assumption after crushing.

At present, Feng et al. [7] believe that the bending stiffness of the segmental joints is usually regarded as a constant, which leads to deviations in the calculated internal force and deformation. As a result, an iterative approach for estimating the bending stiffness of segmental joints was presented, and the sensitivity of the method was assessed. Zhang [8] proposed a model considering shear spring. The deficiency of this model was that the nonlinearity of joint materials, especially concrete, was not fully considered. Lee and Ge [9,10] employed the ‘Force Method’ to determine the internal force and displacement of segmental joints in their investigation of shield tunnel design theory. Other researchers [11,12,13,14] used beam–spring or shell–spring models to study the mechanical properties of joints. Additionally, Li [15] and Qiu [16] proposed two progressive models to simulate all mechanical behaviors of segmental joints successfully.

With improved computer ability, the accuracy, convenience, and low cost of numerical simulation are also highlighted. Many studies [17,18,19,20,21,22] focus on the fine modeling of segments. As for the segmental joints, Oggeri [23] proposed a set of multi-ring finite element (FE) models, which analyzed in detail the segment interaction and stress concentration experienced by the last erected ring, which was simultaneously subjected to the TBM ram loads and the advance of the sealing pressure. Li et al. [24] established an acceptable numerical model for the joints of the cast-iron segment and conducted an experimental test on the mechanical behavior of the joints by the numerical model. Ge et al. [25] proposed a simplified numerical simulation method to modify the local stiffness. At the same time, tunnel information modeling based on building information models has been used [26].

This paper conducts three-dimensional simulations for different axial loads and single- and double-layer linings of large-diameter shield tunnels under high water pressure. The proposed joint bending stiffness mechanical model is used to calculate the corresponding bending stiffness of the joints under different axial forces, concrete strengths, and bolts. The sensitivity of various factors to the stiffness of the segmental joints is analyzed, which provides a reference for the segment design of large-diameter shield tunnels under high water pressure.

## 2. Numerical Simulation of Mechanical Characteristics of Segmental Joints

### 2.1. Modeling

**Figure 1:**
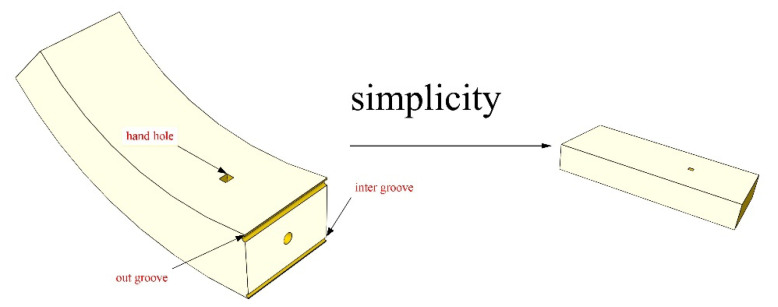
Simplified diagram of segmental joints.

The flat-shaped segment is used instead of the actual arc-shaped segment. In fact, this simplification was used in previous experiments. Because the evident structural response of the joint is restricted to the terminal area, according to Saint-Venant’s principle [27], there is no need to test the whole length of the segment. The detailed geometry of the segmental joint is shown in Figure 1.

The single-layer lining segment model and the double-layer lining segment model were created to investigate lining effects on segments and joints, respectively. Concrete and bolts are employed the SOLID65 and LINK180 elements, respectively. The segment concrete material adopted the empirical analogy method, referring to the 15 m large shield tunnel selection specifications of the Shanghai Chongming Yangtze River Crossing Tunnel. The lining material is C60 concrete, and the mechanical properties are shown in Table 1. In addition, the concrete adopted the multi-linear isotropic hardening model MISO. The thickness of the single-layer lining segment was 650 mm.

The segment ring of the shield tunnel is composed of segments such as the cap block, adjacent block, and standard block, which are generally connected by straight bolts, bent bolts, and oblique bolts (pin-type bolts). However, straight bolts were chosen for simulation in this study due to the convenience of numerical simulation.

The stress–strain uniaxial curve formula of concrete follows.

$\varepsilon_{c}\leq \varepsilon_{0}$:

(1) $$\sigma_{c}=f_{c}\left[1-{\left(1-\frac{\varepsilon_{c}}{\varepsilon_{0}}\right)}^{n}\right]$$

$\varepsilon_{0}<\varepsilon_{c}\leq \varepsilon_{cu}$:

(2) $$\sigma_{c}=f_{c}$$

where n=1.83333, $\varepsilon_{0}=0.00205$, and $\varepsilon_{cu}=0.0033$.

**Figure 2:**
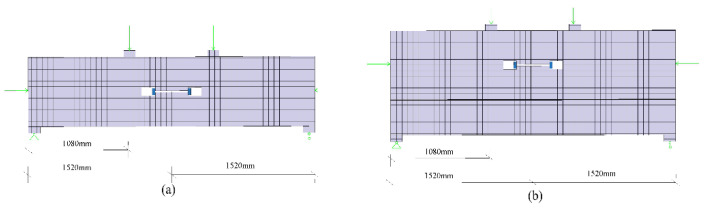
Two-dimensional sketch showing loading of the single-layer lining, (**a**) double-layer lining, and (**b**) segmental joint.

**Figure 3:**
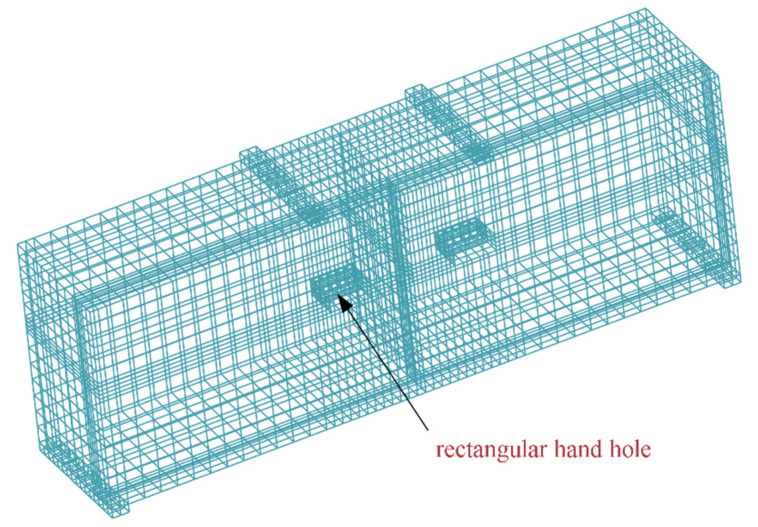
Meshing diagram of the single-layer lining segment.

The size of the rectangular hand hole is 200 mm × 80 mm × 160 mm. The bolt used is an 8.8-grade M24 high-strength bolt with a yield strength of 640 MPa and a diameter of 27 mm. Water and soil pressure is the axial force exerted on the segment. The horizontal axial force loading ranges from 10,000 to 20,000 kN, and the load increment value is 2000 kN. There are six groups of axial-force-loading conditions. The vertical force load increases by 200 kN each time until the concrete of the joints crushes, or the bolt pulls out. The loading diagram of single-layer lining and double-layer lining is shown in Figure 2. Figure 3 shows the meshing diagram of the single-layer lining segment. The parameters of the double-layer lining segments are similar to those of the single-layer lining. However, the thickness of the double-layer lining section exceeds that of the single-layer lining segment by 350 mm.

### 2.2. Comparison of the Destruction Process

**Figure 4:**
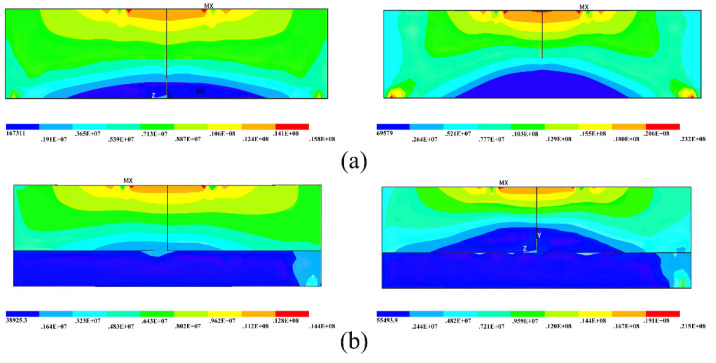
Comparison of the stress failure process between the (**a**) single-layer lining and (**b**) double-layer lining.

**Figure 5:**
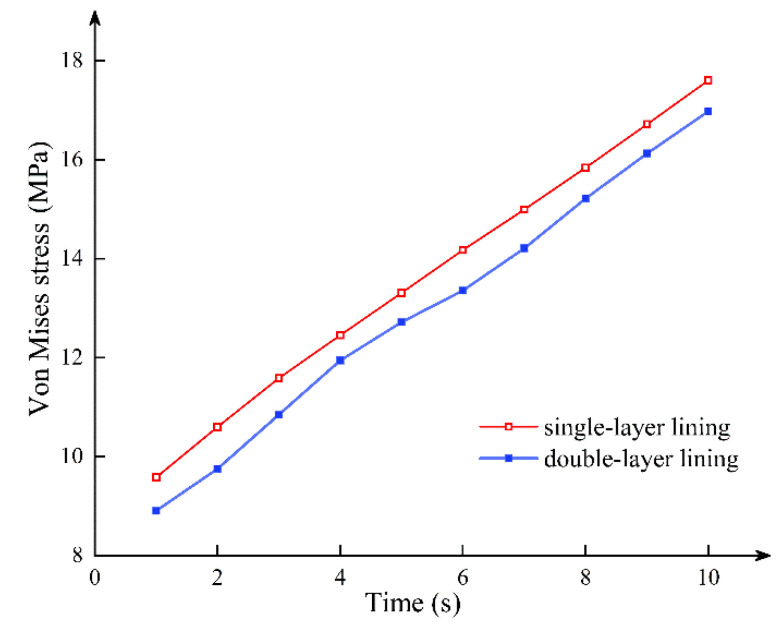
Stress–time curve of single-layer lining and double-layer lining at a node around the concrete compression area.

Figure 4 shows the stress cloud of single-layer lining and double-layer lining segmental joints at the fifth and tenth load steps when the axial force is 10,000 kN. It can be seen that the single-layer lining and the double-lined segmental joint have a similar stress failure process. In other words, from the whole cross-section compression, the bolt is not under force; the compression area continues to move up, and the bolt participates in the force. At that time, the triangular stress zone appears in the lower part of the concrete under the tension of the bolt until the uppermost concrete reaches the yield compressive strength, or the stress concentration at the bolt hand hole exceeds the compressive strength of the concrete. Figure 5 shows the relationship between the stress of a node around the concrete compression zone and the loading time under the two lining modes. Although the double lining increases the thickness of the segment, the improvement of the overall ultimate bearing capacity of the segment is limited. It can be considered that the double-layer lining structure only plays a role in preventing tunnel leakage and lining corrosion. Yan et al. [28] believed that after the surrounding pressure of the shield tunnel is stable, the load is all directly applied to the primary lining segment. Therefore, the primary lining segment does not transfer the external force to the secondary lining without a particular load. That is, the double-layer lining only plays an auxiliary role. However, the conclusions of this paper mainly prove that the secondary lining contributes little to the bearing capacity of the joint and does not mean that the secondary lining has a weak effect on the overall mechanical properties of the shield tunnel.

### 2.3. Stress Concentration at the Hand Hole of the Segmental Joint

**Figure 6:**
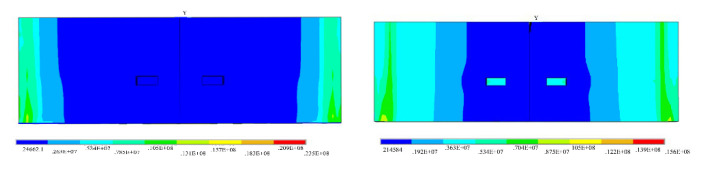
Failure process of the hand hole in the single-layer lining segment.

**Figure 7:**
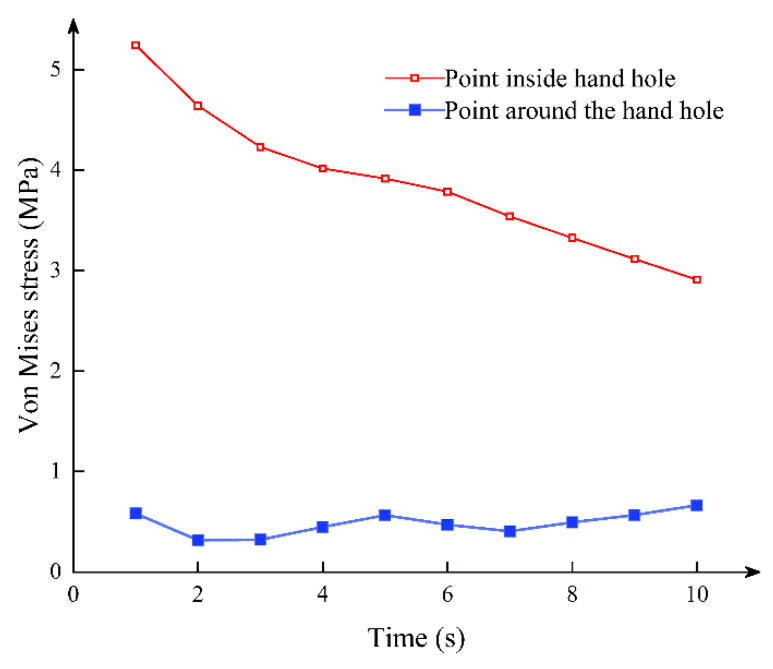
Stress–time curve of internal nodes and surrounding nodes of the hand hole.

Figure 6 shows the stress state of the joint in the fifth and tenth load steps. As shown in Figure 7, in the early stage of stress, the stress of the hand hole internal node is much larger than that of the surrounding node. With the increase of stress time, the stress of the hand hole internal node gradually decreases. During the entire section compression stage, the stress concentration at the hand hole is large because of the high axial pressure. In addition, an extrusion effect can be seen in the stress condition. The concrete on one side of the joint bolt is opened as the positive bending moment increases, reducing the stress concentration effect of the hand hole.

**Figure 8:**
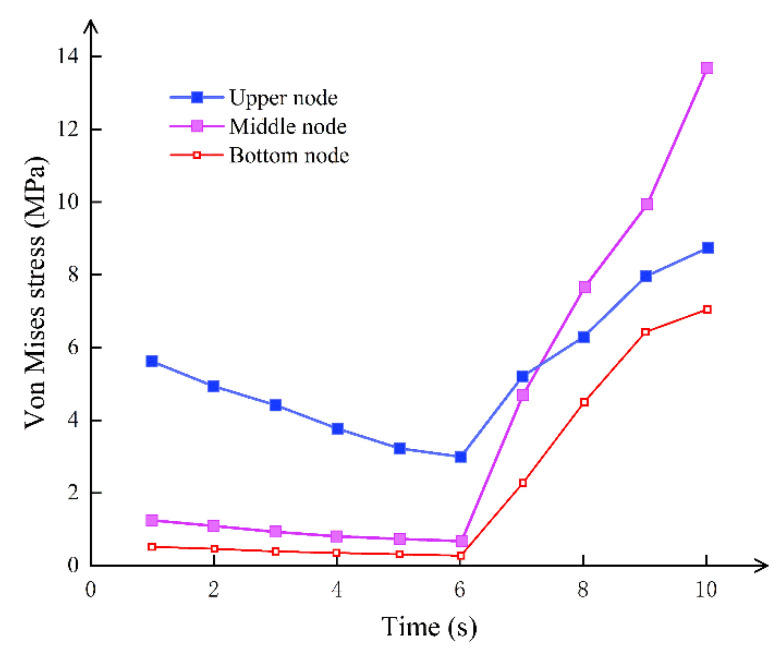
Stress–time curve of the upper, middle, and lower joints on the interface between the bolt and hand hole.

The stress concentration state of the contact surface between the bolt and the hand hole is precisely the opposite of the hand hole. When the bolt is not stressed, the stress on the contact surface of the bolt and the hand hole is greater than the stress on the middle and lower nodes, as shown in Figure 8. The stress is centered on the contact surface at this moment, which is mostly due to the geometry of the hand hole. The node stress rapidly grows once the bolt joins the force until it exceeds the stress concentration effect given by the hole form. The tensile force of the bolt increases with time, forcing the concrete on the bolt contact surface and the concrete on the compression side to yield simultaneously. In the process of designing the shield tunnel with high water pressure, multiple rows of bolts or contact surfaces large enough should be used to ensure that the stress is concentrated in the controllable range while balancing the weakening effect of the size of the hand hole on the segmental joint.

### 2.4. Crack Development of the Segmental Joint

**Figure 9:**
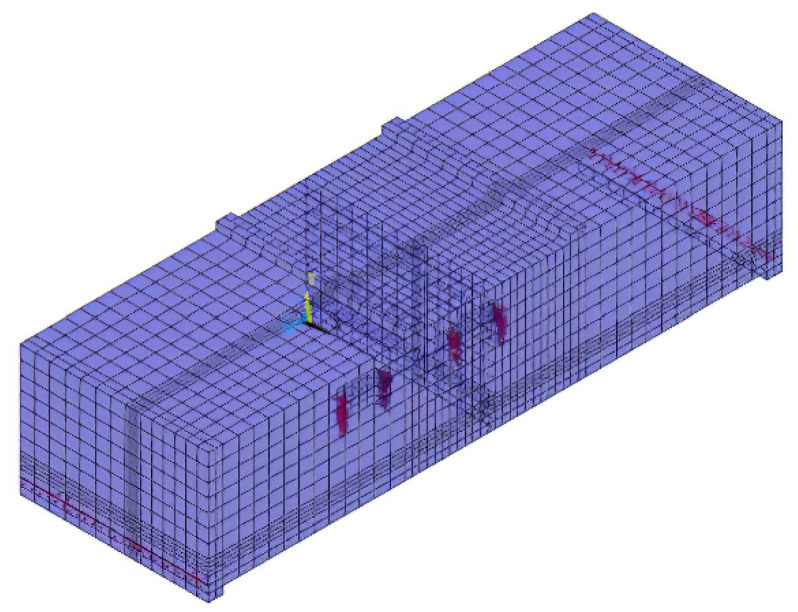
Crack development at the initial stage of joint stress.

**Figure 10:**
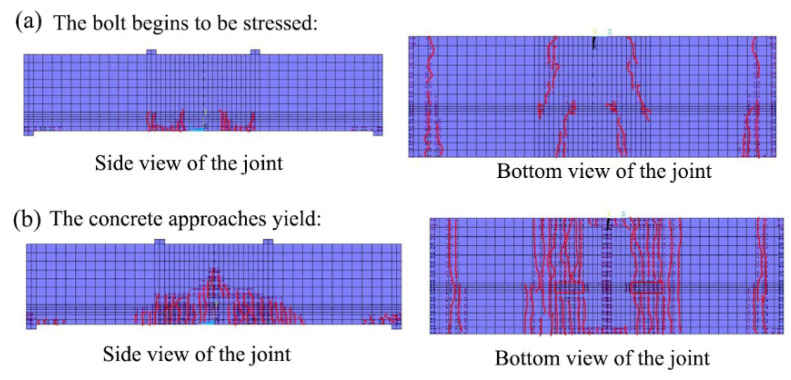
Crack development near the concrete hand hole segment.

Figure 9 shows the formation of concrete cracks under an axial force of *N* = 10,000 kN. At the beginning of the joint under force, the joint cracks first emerged at the point of stress concentration and extended lengthwise from both ends of the short side of the hand hole. Because of the restriction effect at the support, lateral cracks appeared outside the section. With the gradual transfer of the stress concentration area after the segmental joint is opened and the bolt begins to bear the strain, the form of the crack also changes. The vertical cracks generated by the pulling force of the hand hole begin to form on the side of the joint as the tension carried by the bolt increases, as shown in Figure 10a. Cracks extending to the edge of the segment appear at both ends of the hand hole, and the development direction of the crack is n t perpendicular to the side of the segment but exhibits an angle of about 30 degrees. As shown in Figure 10b, before the concrete in the compression area is close to yielding, the bolt has a huge stress concentration effect on the concrete, leading to tensile failure of the concrete near the hand hole. The longitudinal crack is localized around the hand hole and propagates to the bottom of the joint.

### 2.5. Experimental Verification

**Figure 11:**
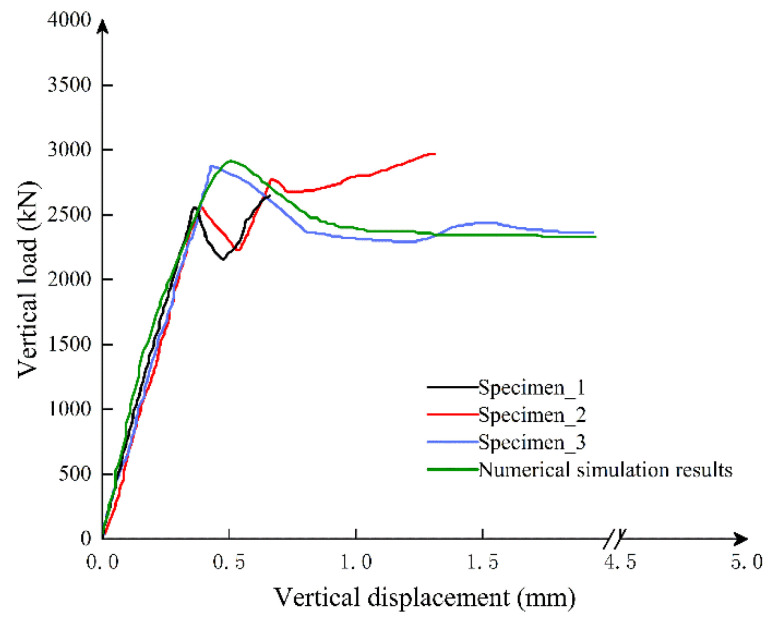
The load–displacement curve of a test piece for the straight bolt and rectangular hand hole joint.

The crack distribution in simulation is consistent with the scaled experiment test by Wang et al. [29]. Figure 11 shows that under different axial forces, the displacement curve of the numerical simulation is basically consistent with the displacement measured in the actual experiment, which proves the reliability of the numerical model.

The specimen is the straight bolt with rectangular hand hole test piece in the experiment. In the numerical simulation, the peak load and peak displacement of the straight bolt and rectangular hand hole specimen are larger than the actual test. There are two reasons that may explain this observation:

(1) In the test, the concrete specimen often spalls in a large area when it reaches about 80% of the peak load. However, this factor is not considered in the numerical simulation, which causes the peak load to be too large.

(2) The randomness and variability of the concrete specimen itself, and the peak load and peak displacement in the test are the averages of the three specimens. However, the randomness and variability of the concrete specimen itself were not considered in the numerical simulation.

### 2.6. Joint Bending Stiffness under Different Axial Forces

**Figure 12:**
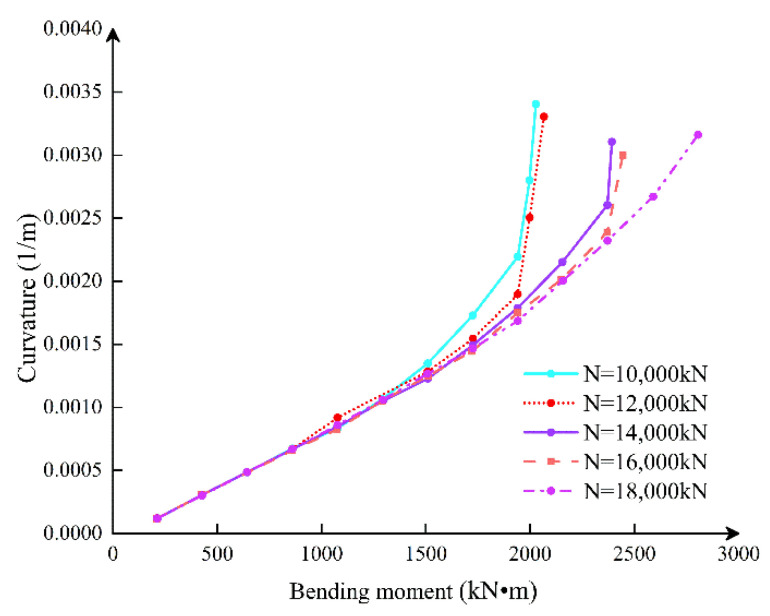
*M–ϕ* curves of joint under different axial pressures.

The *M–ϕ* curves of joints under different axial pressures are shown in Figure 12. The critical moment of the joint opening increases with increasing axial force, but bolt deformation reduces with increasing axial force. Compared with N = 10,000 kN and N = 20 000 kN, it is obvious that the ductility of the joint decreases gradually. In terms of the flexural bearing capacity of joints, when the joint has not been opened, the joint stiffness is basically independent of the axial force, which is mainly determined by the nature of the section. However, the magnitude of the axial force determines the critical bending moment when the joint is opened. When the axial force is small, the decreased curvature of the joint flexural rigidity is more remarkable than when the axial force is large, which shows that the increase of the axial force can improve the bending mechanical performance of the joint.

## 3. Theoretical Analysis of Mechanical Characteristics of Single-Layer Lining Segmental Joints

### 3.1. Assumptions

(1) The deformation of the segmental joint conforms to the plane section assumption without considering the influence of the hand strain and the bolt disengagement factors.
(2) The nonlinear constitutive equation of concrete can be linearly simplified by the equivalent principle of deformation energy.
(3) The influence of elastic liner, tenon-grooves interface, and locating block on joint mechanical behavior are ignored.
(4) According to the numerical analysis of the three sections, the high water pressure shield tunnel adopts the single-layer lining.
(5) The bolt is under tension without compression.

The constitutive relation curve of concrete follows:

(3) $$\sigma_{c}=\{\begin{array}{cc} E_{x0}\varepsilon_{x0} & \varepsilon_{c}\leq \varepsilon_{x0}\\ f_{c} & \varepsilon_{x0}<\varepsilon_{c}\leq \varepsilon_{cu} \end{array}.$$

When the strength grade of concrete is less than or equal to *C*50, $\varepsilon_{0}=0.002$ and $\varepsilon_{x0}=0.00133$. For concrete with strength grade *C*60, the correlation coefficient needs to be modified using the strain energy principle. The modified formula is:

(4) $$\varepsilon_{0}=0.002+0.5\left(f_{cu}-50\right)\times 10^{-5}.$$

where $\varepsilon_{0}=0.00205$, $\varepsilon_{x0}=0.00145$, and $E_{x0}=1.9\times 10^{-4}N\cdot mm^{-2}.$

### 3.2. Theoretical Model

#### 3.2.1. Bending Stiffness of the Segmental Joint under Positive Bending Moment

**Figure 13:**
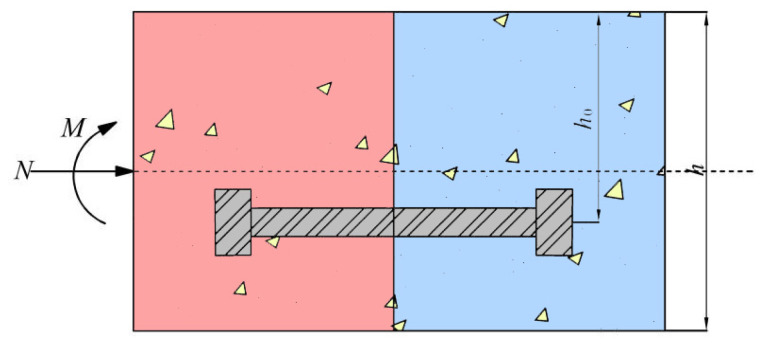
Stress analysis model of the segmental joint (positive bending moment).

In Figure 13, the shield segmental joints are mainly composed of concrete and bolts as the main force-bearing components, subjected to the combined axial force and bending moment in the soil layer; *h_0_* is the distance from the segment’s compression zone edge to the bolt’s centroid, and *h* is the segment’s section height.

The segments of a large-diameter deep-buried shield tunnel present small eccentric compression. In this situation, concrete failure in the compression zone of the segmental joint under positive bending moment is generally earlier than the bolts. Therefore, the joint from stress to failure can be assumed at four stages and the secant method to calculate bending stiffness applies. The bolts in the segmental joints are simplified to elastic–ideal plastic materials. The bending stiffness of the joint under different stress states follows:

(5) $$k_{\theta }=\frac{dM}{d\phi }\approx \frac{\Delta M}{\Delta \phi }.$$

where

(6) Δϕ=Δε⋅l/h.

Stage 1: Whole section in compression.

**Figure 14:**
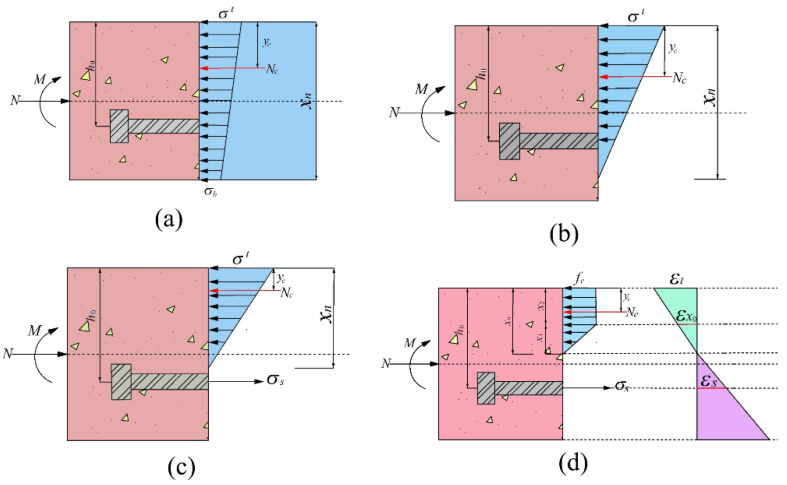
(**a**) First-stage stress-analysis model of the segmental joint $x_{n}=h$. (**b**) Second-stage stress-analysis model of the segmental joint ($h_{0}<x_{n}<h$ ). (**c**) The third-stage stress-analysis model of the segmental joint $0<x_{n}<h_{0}$. (**d**) Fourth-stage stress-analysis model of concrete after yielding.

As shown in Figure 14, the stress pattern of concrete in the compression zone is replaced by an equivalent trapezoidal or triangular stress pattern. The condition is that the magnitude of compressive stress resultant force is equal, and the position of the resultant force is identical; *σ^t^* is the compressive stress at the lower part of the segmental joint, and *σ_b_* is the compressive stress at the upper part of the segmental joint. *Nc* is the resultant force of the concrete under compression, and y*_c_* is the distance from the point of action of the resultant concrete force to the upper edge of the segmental joint.

Simplified balance equation:

(7) $$N=\frac{1}{2}\left(\sigma_{t}+\sigma_{b}\right)bh$$

The action point of resultant force:

(8) $$y_{c}=\frac{2\sigma_{b}+\sigma_{t}}{3\left(\sigma_{b}+\sigma_{t}\right)}h$$

(9) $$M=\frac{1}{2}\left(\sigma_{t}+\sigma_{b}\right)bh\cdot \left(\frac{h}{2}-y\right)$$

Combining Equations (8) and (9):

(10) $$\sigma_{t}=\frac{1}{bh}\left(N+\frac{6M}{h}\right)$$

(11) $$\sigma_{b}=\frac{1}{bh}\left(N-\frac{6M}{h}\right)$$

According to the plane section assumption: $\varepsilon_{b}=\frac{\sigma_{b}}{E_{x0}}$, $\varepsilon_{t}=\frac{\sigma_{t}}{E_{x0}}$.

(12) $$\phi =\frac{\varepsilon_{t}-\varepsilon_{b}}{h}=\frac{12M}{bh^{3}E_{x0}}$$

The boundary conditions to be satisfied in this stage are $0\leq \sigma_{t}\leq f_{c}$, $0\leq \sigma_{b}\leq f_{c}$, $0<\varepsilon_{t}<\varepsilon_{x0}$, and $0<\varepsilon_{b}<\varepsilon_{x0}$. In addition, when $\sigma_{t}>f_{c}$, the concrete is directly crushed, and the segment loses its bearing capacity.

Stage 2: The joint is opened, but the bolt is not forced.

The stress of concrete in the compression zone is simplified to a triangular stress distribution. At this stage, the concrete is always in the elastic stage.

From equilibrium equations:

(13) $$\frac{1}{2}\sigma_{t}x_{n}b=N.$$

The bending moment:

(14) $$\frac{1}{2}\sigma_{t}x_{n}b\left(\frac{h}{2}-\frac{x_{n}}{3}\right)=M.$$

Combining Equations (13) and (14):

(15) $$\phi =\frac{\varepsilon_{t}}{x_{n}}=\frac{2M}{9b{\left(\frac{h}{2}-\frac{M}{N}\right)}^{2}E_{x0}}.$$

The boundary conditions to be satisfied in this stage are: $\varepsilon_{t}\leq \varepsilon_{x0}$, $h_{0}\leq x_{h}<h$, and $\sigma_{t}\leq f_{c}.$ Stage 3: The bolts begin to bear tension, the compression zone height $x_{n}<h_{0}$, and the concrete is still in the elastic compression state.

The equilibrium equation of force follows:

(16) $$\frac{1}{2}b\sigma_{t}x_{n}=\sigma_{s}A_{s}+N$$

(17) $$M=\frac{1}{2}b\sigma_{t}x_{n}\left(h_{0}-\frac{1}{3}x_{n}\right)-N\left(h_{0}-\frac{h}{2}\right).$$

According to the plane section supposition:

(18) $$\sigma_{s}=\frac{E_{s}}{E_{x0}}\left(\frac{h_{0}-x_{n}}{x_{n}}\right)\sigma_{t}.$$

Let $\alpha_{s}=\frac{E_{s}}{E_{x0}}$ Simultaneously, solve the Equation (19).

(19) $$Ax_{n}^{3}+Bx_{n}^{2}+Cx_{n}+D=0$$

(20) $$\left\{\begin{array}{c} A=\frac{1}{3}bN\\ B=b(M-\frac{Nh}{2})\\ C=2\alpha_{s}[M+N(h_{0}-\frac{h}{2})]A_{s}\\ D=-2\alpha_{s}h_{0}[M+N(h_{0}-\frac{h}{2})]A_{s} \end{array}\right.$$

The curvature is obtained from $\phi =\frac{\varepsilon_{t}+\varepsilon_{s}}{h_{0}}$, and then the bending stiffness of the joint is obtained from Equation (5).

The boundary conditions are: $0\leq \sigma_{c}<f_{c}$, $0<\sigma_{s}<f_{sy}$, $0<\varepsilon_{t}<\varepsilon_{x0}$, $0<\varepsilon_{s}<\varepsilon_{y}$, and $0<x_{n}<h_{0}$.

Stage 4: Concrete yielded in the compression zone at the segmental joint; bolt did not yield.

(21) $$\frac{1}{2}f_{c}bx_{1}+f_{c}bx_{2}=\sigma_{s}A_{s}+N$$

(22) $$x_{1}=\frac{\varepsilon_{x0}}{\varepsilon_{t}}x_{n}$$

(23) $$x_{2}=\left(1-\frac{\varepsilon_{x0}}{\varepsilon_{t}}\right)x_{n}$$

where $x_{1}$ is concrete height in the unyielding compression zone and $x_{2}$ is the height in the yielding compression zone.

Combining Equations (21)–(23):

(24) $$\sigma_{s}=\frac{f_{c}bx_{n}\left(1-\frac{1}{2}\frac{\varepsilon_{x0}}{\varepsilon_{t}}\right)-N}{A_{s}}.$$

Because $\sigma_{s}=E_{s}\varepsilon_{s}=E_{s}\left(\frac{h_{0}-x_{n}}{x_{n}}\right)\varepsilon_{t}$, eliminate $\sigma_{s}$:

(25) $$f_{c}\left(1-\frac{1}{2}\frac{\varepsilon_{x0}}{\varepsilon_{t}}\right)x_{n}^{2}+\left(E_{s}\varepsilon_{t}A_{s}-N\right)x_{n}-E_{s}\varepsilon_{t}A_{s}h_{0}=0.$$

The iterative solution can solve for the concrete height $x_{n}$ in the compression zone under different maximum concrete strain conditions.

The action point of resultant force follows:

(26) $$y_{c}=\frac{\frac{1}{2}[x_{1}(x_{2}+\frac{1}{3}x_{1})+x_{2}^{2}]}{\left(x_{2}+\frac{1}{2}x_{1}\right)}.$$

When the maximum strain of concrete at the segmental joint is $\varepsilon_{t}$, the corresponding bending moment *M* is:

(27) $$M=\sigma_{s}A_{s}\left(h_{0}-y_{c}\right)-N\left(\frac{h}{2}-y_{c}\right).$$

The curvature is obtained from $\phi =\frac{\varepsilon_{t}+\varepsilon_{s}}{h_{0}}$, and then the bending stiffness of the joint is obtained from Equation (5).

The boundary conditions are $0<\sigma_{s}<f_{sy}$, $\varepsilon_{x0}<\varepsilon_{t}<\varepsilon_{cu}$, $0<\varepsilon_{s}<\varepsilon_{y}$, and $0<\varepsilon_{s}<\varepsilon_{y}$.

#### 3.2.2. Bending Stiffness of the Segmental Joint under Negative Bending Moment

Under the negative bending moment, the shield segmental joint experiences two stages. Since the derivation process is similar to the positive bending moment, the negative bending moment derivation process is omitted.

Stage 1: Whole section in compression.

The curvature of the section under *M* and *N* loads is:

(28) $$\phi =\frac{\varepsilon_{t}-\varepsilon_{b}}{h}=\frac{12M}{bh^{3}E_{x0}}.$$

The boundary conditions to be satisfied in this stage are: $0\leq \sigma_{b}\leq f_{c}$, $0\leq \sigma_{t}\leq f_{c}$, $0<\varepsilon_{t}<\varepsilon_{x0}$, and $0<\varepsilon_{b}<\varepsilon_{x0}$.

Stage 2: The top of the joints is open, and the concrete is still elastic.

The relationship between curvature and bending moment is:

(29) $$\phi =\frac{\varepsilon_{t}}{x_{n}}=\frac{2N}{9ab{\left(\frac{h}{2}-\frac{\left|M\right|}{N}\right)}^{2}E_{x0}}.$$

The boundary conditions to be satisfied in this stage are: $\varepsilon_{b}\leq \varepsilon_{x0}$ and $\sigma_{b}\leq f_{c}$.

## 4. Discussion

### 4.1. Verification

**Figure 15:**
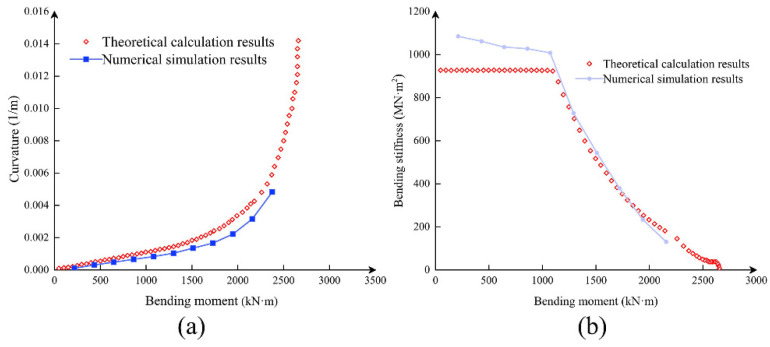
Comparison of *M–ϕ* (**a**) and *K_θ_–M* (**b**) curves between the theoretical calculation and numerical simulation (*N* = 10,000 kN, positive bending moment).

Figure 15 shows that the numerical simulation results are relatively close to the theoretical calculation, which proves the reliability of the theoretical calculation results. The elastic modulus of the concrete in the initial stage of numerical simulation is greater than the equivalent elastic modulus used in the mathematical derivation. The curvature value error of the theoretical calculation result is more significant in the *M–ϕ* curves. From the perspective of practical engineering, the theoretical calculation is safer. The mechanical parameters of segmental materials are shown in Table 2.

### 4.2. Influence of Axial Load on Bending Stiffness of the Segmental Joint

**Figure 16:**
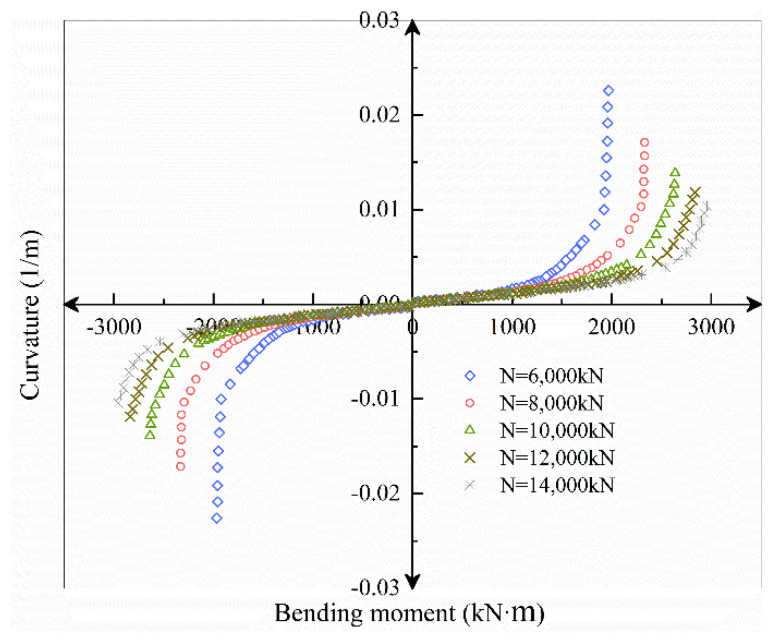
*M–ϕ* relationship curve under different axial forces.

**Figure 17:**
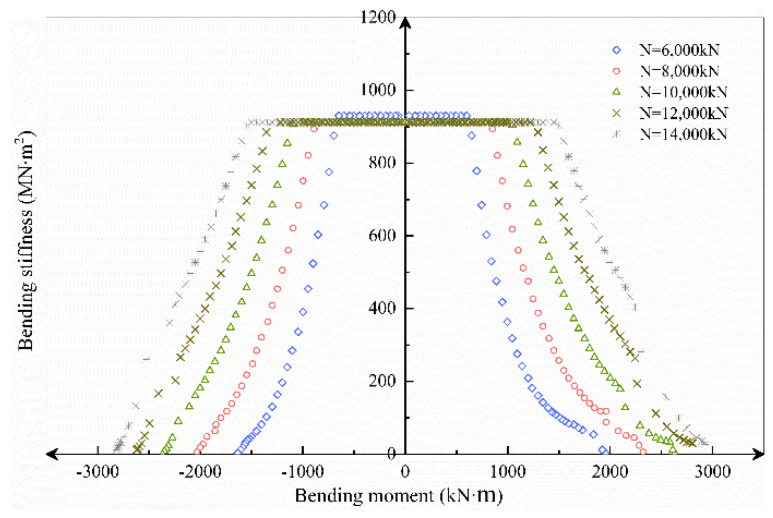
*K_θ_–M* curves under different axial forces.

**Figure 18:**
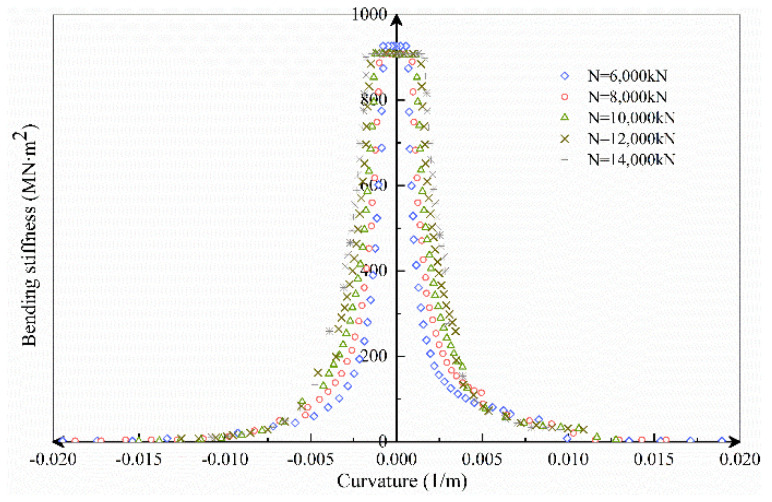
*K_θ_–ϕ* curves under different axial forces.

The axial load range selected in this section is *N* = 6000~14,000 kN, increasing at a rate of 2000 kN. According to the axial load, *M–ϕ*, *K_θ_–M*, *K_θ_–ϕ* and curves under different axial force conditions are obtained by the theoretical analysis model, as shown in Figure 16, Figure 17 and Figure 18.

Figure 16 shows that as the axial force increases, regardless of whether the bending moment is positive or negative, the joint opening under the same bending moment condition decreases, while the ultimate bending moment capacity increases. Nonetheless, the joint’s ultimate deformation continues to diminish, bringing it closer to brittle failure. In the process of increasing the axial force from 6000 to 8000 kN, the ultimate bearing capacity of the positive bending moment increased by 19%, the ultimate bearing capacity of negative bending moment increased by 24%; the ultimate curvature of positive bending moment decreased by 23%, and the ultimate curvature of negative bending moment decreased 27%. In the process of increasing the axial force from 12,000 to 14,000 kN, the ultimate bearing capacity of the positive bending moment increased by 4%, while the negative bending moment increased by 7%; the positive bending moment limit curvature decreased by 12%, and the negative bending moment decreased by 14%.

It can be seen that the ultimate bearing capacity and ultimate deformation under negative bending moment conditions are more sensitive to the increase of axial force. At the same time, with the increase of axial force, the ultimate bearing capacity and ultimate deformation both decrease nonlinearly.

Figure 17 shows that, for the same external moment load, as the axial force increases, the flexural stiffness and ultimate moment bearing capacity rise proportionally, while the bolt resistance increases and the height of the concrete compression area decreases. Finally, the effect of axial force on bending stiffness steadily diminishes.

Figure 18 illustrates that the larger the axial force, the greater the bending stiffness when the bolt participates in the force early. As the bolt continued to force, the growth of the curvature was restricted by the bolt. The curvature changes are about the same under varied axial force situations, indicating the importance of the bolt in limiting joint deformation. Furthermore, in the first, second, and third stages, the greater the axial force, the greater the bending stiffness of the joint. After the bolt yielded, however, it was easier for brittle failure to occur.

### 4.3. Influence of Concrete Strength on Bending Stiffness of the Segmental Joint

*C*50 and *C*70 concrete with compressive strength $f_{c}=23.1\;N\cdot {\mathrm{mm}}^{-2}$ and $f_{c}=31.8\;N\cdot {\mathrm{mm}}^{-2}$ were selected for comparative study.

**Figure 19:**
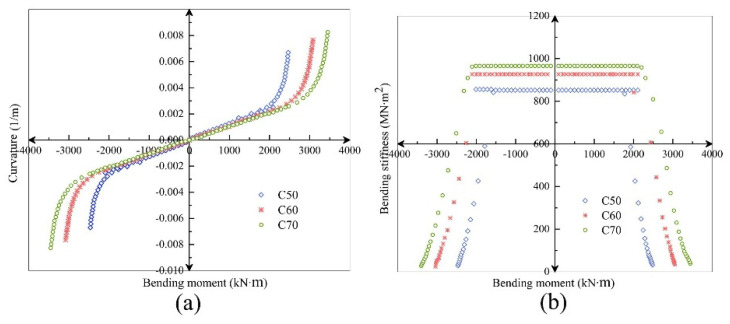
Comparison of *M*–*K* (**a**) and *K_θ_–ϕ* (**b**) relationship curves of *C*50/*C*60/*C*70 segmental joints (*N* = 20,000 kN).

It can be seen from Figure 19 that under the condition of large axial force, the bending stiffness also increases with the increase of concrete strength. Before the joint is opened, the concrete strength has little effect on the bending stiffness; after the joint is opened, the influence of the concrete strength on the bending stiffness is significantly increased, especially for the joint stiffness from *C*50 to *C*60. The influence of increased concrete strength is diminished again as bolt tension increases. In conclusion, the sensitivity of the influence of the increase of concrete strength on the flexural bearing capacity and flexural stiffness of the joint is positively correlated.

### 4.4. Influence of Bolt Strength on the Bending Stiffness of the Segmental Joint

**Figure 20:**
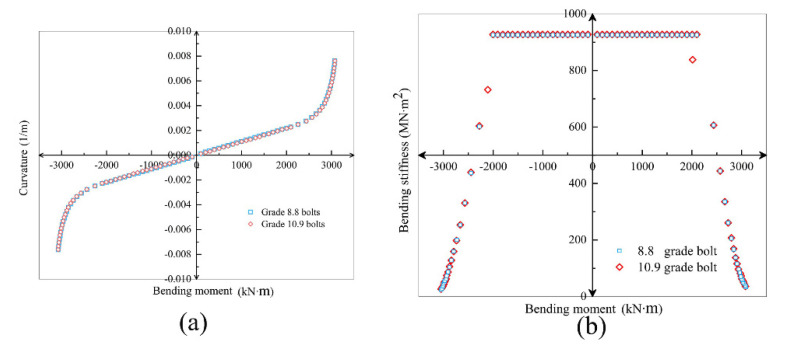
Comparison of *M–ϕ* (**a**) and *K_θ_–ϕ* (**b**) curves of grade 8.8 and 10.9 bolts (*N* = 20,000 kN).

Select the same diameter M24 bolt, and the strength grade is 10.9 and 8.8. The comparison of the calculated results is as Figure 20.

Figure 20 shows that the increase in bolt strength has little effect on bending stiffness and deformation. The influence of the bolt strength is even smaller than the influence of the bolt diameter. Under high axial force, the contribution of bolt strength to the segmental joint is correspondingly weakened.

## 5. Conclusions

The numerical simulation of the segmental joint provides a basis for the theoretical analysis of joint bending stiffness. The theoretical model of the bending stiffness of the segmental joint proposed in this paper can reflect its bending performance well.

(1) In terms of bearing capacity, the secondary lining has limited effect on improving the bearing capacity of the joint, which is not used as a bearing structure.

(2) Under the condition of large axial force, the axial force gradually changes from improving the joint bearing capacity to causing joint failure.

(3) The ultimate bearing capacity and bending stiffness increased by concrete strength are also affected by the strength of the bolts. Therefore, if high-strength concrete is used, it is necessary to consider matching reasonable high-strength bolts to avoid having the bolt yielding first.

(4) Increasing concrete strength significantly improves the overall flexural bearing capacity and stiffness of the segmental joint. In contrast, bolt diameter and bolt strength have little effect on the flexural bearing capacity and stiffness of the joint, especially under large axial force.

(5) The failure process and crack development of the segmental joint model in the numerical simulation are consistent with the experimental data, which proves its reliability.

## Figures and Tables

**Table 1:** Mechanical parameters of C60 concrete.

Symbol	Description	Value
*f_c_*	uniaxial compressive strength	27.5 MPa
*f_t_*	uniaxial tensile strength	2.04 MPa
*β_t_*	shear transfer coefficient of gaping fissure	0.5
*β_c_*	shear transfer coefficient of closed cracks	0.95
*E*	elastic modulus	3.6 × 10^4^ MPa
ν	Poisson ratio	0.2
*T_c_*	tensile stress release coefficient	0.6

**Table 2:** Mechanical parameters of the segment material.

Segment		Bolt		Concrete	
Width b/mm	Thickness h/mm	Diameter /mm	Strength level	Label	The design value of compressive strength $f_{c}/N\cdot mm^{-2}$
2000	650	27	8.8	C60	27.5

## Data Availability

Not applicable.

## Abbreviations

*ε*_0_	Yield compressive strain of concrete
*ε_cu_*	Ultimate compressive strain of concrete
*ε_x_*_0_	Strain energy equivalent to yield compressive strain of concrete
*ε_s_*	Bolt strain
*ε_sy_*	Bolt yield strain
*E_x_*_0_	Strain energy equivalent concrete elastic modulus
*E_s_*	Bolt elastic modulus
*x_n_*	Height of compression zone of joint concrete
*h*	Segment height, 650 mm
*b*	Segment width, 2000 mm
*h*_0_	Distance from bolt center to concrete far from bolt side
*ϕ*	The curvature of the joint under bending moment and axial force
*σ_t_*	Concrete stress on the edge away from the bolt
*σ_b_*	Concrete stress near the edge of the bolt
*ε_t_*	Concrete strain on the edge away from the bolt
*ε_b_*	Concrete strain near the edge of the bolt
*f_c_*	Design value of concrete yield strength
*f_sy_*	Design value of bolt tensile strength
*σ_s_*	Bolt stress
*σ_c_*	Concrete stress
*A_s_*	Total cross-sectional area of joint bolts
